# Erratum: Quantitative analysis of magnetic spin and orbital moments from an oxidized iron (1 1 0) surface using electron magnetic circular dichroism

**DOI:** 10.1038/srep15590

**Published:** 2015-10-29

**Authors:** Thomas Thersleff, Jan Rusz, Stefano Rubino, Björgvin Hjörvarsson, Yasuo Ito, Nestor J. Zaluzec, Klaus Leifer

Scientific Reports 5: Article number: 13012; 10.1038/srep13012published online: 08172015; updated: 10292015

In the original version of this Article, Klaus Leifer was incorrectly listed as being affiliated with ‘Department of Physics and Astronomy, Uppsala University, Uppsala, Sweden’. The correct affiliation is listed below:

Department of Engineering Sciences, Division of Applied Materials, Uppsala University, Uppsala, Sweden.

This error has now been corrected in both the HTML and PDF versions of the Article.

