# Supplementary material for: Quantitative analysis of magnetic spin and orbital moments from an oxidized iron (1 1 0) surface using electron magnetic circular dichroism
Source: Sci Rep. 2015 Aug 17;5:13012. doi: 10.1038/srep13012 (PMC4538391; doi:10.1038/srep13012)
Supplement: Supplementary Information [file srep13012-s1.pdf]

Supplementary Information: Quantitative analysis of magnetic spin and orbital moments from an oxidized iron (1 1 0) surface using electron magnetic circular dichroism

Thomas Thersleff*[1], Jan Rusz[2], Stefano Rubino[1,3], Björgvin Hjörvarsson[2], Yasuo Ito[4], Nestor Zaluzec[5] and Klaus Leifer†[1]

[1]Department of Engineering Sciences, Uppsala University, Uppsala, Sweden
[2]Department of Physics, Uppsala University, Uppsala, Sweden
[3]Department of Physics, University of Oslo, Oslo, Norway
[4]Department of Physics, Northern Illinois University, DeKalb, IL, USA
[5]Electron Microscopy Center, Argonne National Laboratory, Argonne, IL, USA

ABSTRACT

Supplementary information for the manuscript "Quantitative analysis of magnetic spin and orbital moments from an oxidized iron (1 1 0) surface using electron magnetic circular dichroism."

*Corresponding author. Email: thomas.thersleff@angstrom.uu.se
†Corresponding author. Email: klaus.leifer@angstrom.uu.se

Spectral Moiré analysis of the cladding oxide layers

The analysis of the structure of the oxide cladding layers involves a detailed discussion of Moiré phenomena, which is presented here. We present our reasoning for describing the oxide layer as $Fe_{3-\delta}O_4$ based on structural and EELS arguments.

Iron is known to oxidize very rapidly in the presence of oxygen, and its oxidation process has been extensively studied.[1–5] For an Fe (1 1 0) surface, under the presence of oxygen saturation and at room temperature conditions, it has been shown that the most stable oxide surface layer is Magnetite (Fe_3O_4).[3,4] Other potential oxide phases that can form include Hematite (Fe_2O_3),[2] Wüstite (FeO), iron-deficient Wüstite ($Fe_{1-x}O$),[1] and Maghemite (γ-Fe_2O_3). Finally, it is important to point out that it is possible to form a disordered variation of these oxides of the form $Fe_{3-\delta}O_4$, where δ varies between 0 (Fe_3O_4) and 0.33 (γ–Fe_2O_3).[6] Each of these phases will be considered in this section.

Despite the rapid oxidation process, the majority of the film has not been completely oxidized in the direction of the electron beam propagation. This conclusion can be drawn from the data presented in figures 2 and 3 of the main text. In figure 2, there is no known single crystal structure that can uniquely describe the observed symmetry operations, specifically the three-fold reflections that appear at regular intervals. Moreover, in figure 3b, the lowest frequency is 0.98 nm^{-1}. This is observed in the real-space image as a fringe of spacing 1.02 nm, which is a lattice spacing that is not present in the iron oxide systems. It can thus only arise as a consequence of dynamical scattering between two overlapping crystals.

To fully understand the data presented in figure 2 and 3, it is necessary to identify the two overlapping crystal phases. A reasonable assumption is that one of these phases is metallic Fe, since this was the starting material. Indeed, Bragg reflections that match closely to metallic Fe in the [1 1 0] direction are observed for both the CBED pattern as well as the Fourier transformed HRTEM image, and these are indexed appropriately in the figures. The second phase is thus most likely to be one of the iron oxides mentioned above. According to the spectral analysis of Moiré contrast, the full geometric transmittance function of this second phase must also be present within the experimental data.[7] The only iron oxide phases that would fit some of the remaining reflections are FeO, γ-Fe_2O_3, and Fe_3O_4. Hematite does not fit and can be excluded at this stage.

The final determination of phases can be made by simulating the power spectra for each of the oxide materials overlapped with metallic iron in the direction of the electron beam. The superposition of the transmittance functions of crystal structures $r_i(x,y)$ in the image domain is described by a multiplicative model

$$r(x,y) = \prod_i r_i(x,y). \tag{1}$$

The Fourier transform of this product can be described as the convolution of the Fourier transform of the individual transmittance functions $R_i(u,v)$

$$R(u,v) = \mathfrak{F}[r(x,y)] = R_1(u,v) \otimes R_2(u,v) \otimes \cdots \otimes R_i(u,v). \tag{2}$$

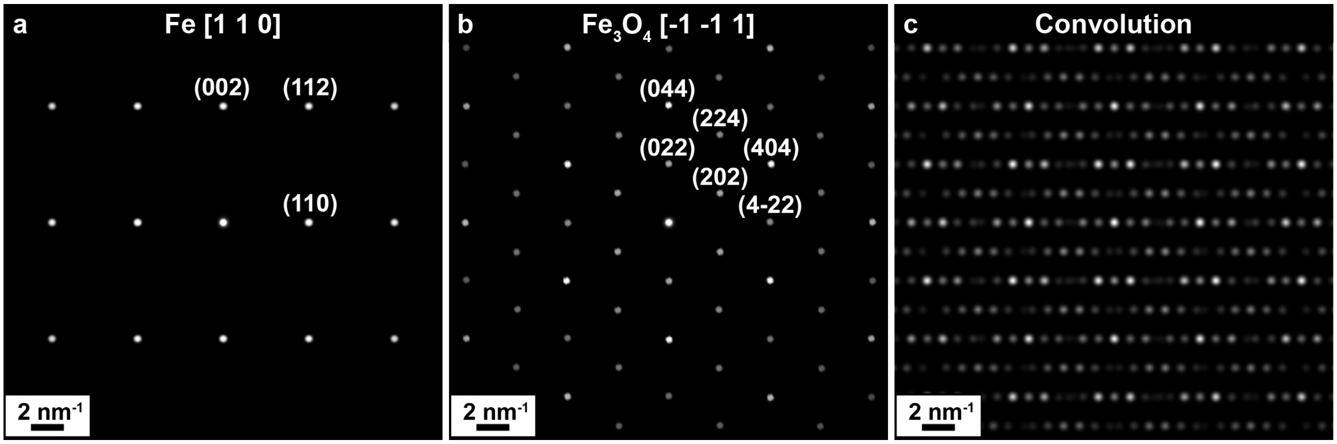

Figure 1. (a) $R_{\mathrm{Fe}}(u,v)$ in the [1 1 0] direction. (b) $R_{\mathrm{Magnetite}}(u,v)$ in the $[\bar{1}\ \bar{1}\ 1]$ direction. (c) Convolution of (a) and (b) ($R(u,v)$).

We can make use of this property to simulate the expected power spectrum $R(u,v)$ generated between metallic iron (defined here as $R_{\mathrm{Fe}}(u,v)$) superposed with the oxide materials listed above, subsequently comparing it with experiment to assess the

most likely explanation. This simulation is demonstrated figure S1. $R_{\mathrm{Fe}}(u,v)$ in the [1 1 0] direction and $R_{\mathrm{Magnetite}}(u,v)$ in the [1 1 1] direction were calculated using the Java EMS (JEMS) software package[8] and are shown in figures S1a and S1b, respectively. The intensities of the Bragg reflections have also been estimated based on the thicknesses of the materials and Peng-Ren-Dudarev-Wehlan (PRDW) atomic form factors.[9] Note that the damping effect of the contrast transfer function of the objective lens system is not considered in the simulations, which explains the presence of Bragg reflections up to higher orders than in the experimental data.

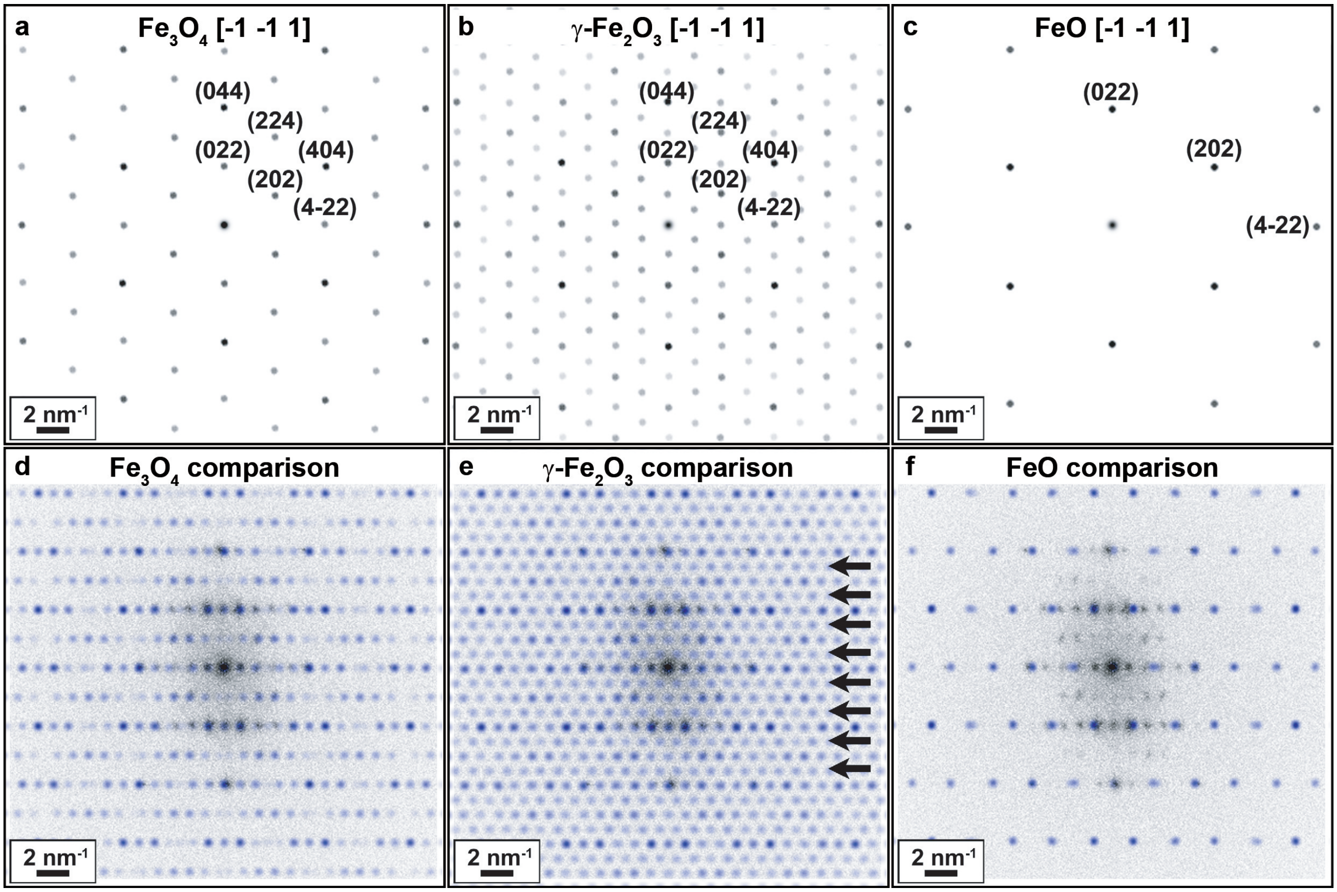

Figure 2. At top, the simulated transmittance functions for Magnetite $[\bar{1}\ \bar{1}\ 1]$, Maghemite $[\bar{1}\ \bar{1}\ 1]$, and Wüstite $[\bar{1}\ \bar{1}\ 1]$ are presented in (a), (b), and (c), respectively. Their convolutions with Fe [1 1 0] are presented in (d), (e), and (f), respectively. The convolved data are overlaid on top of the Fourier-tranformed HRTEM image with the same scale, and (d) clearly demonstrates the best fit between theory and experiment. Non-observed "rows" of reflections are denoted with white arrows in (e). Note that the contrast is inverted to ease visual inspection.

The convolution spectra of all of the possible oxide fits are compared to the reflections observed in the Fourier transformed HRTEM image in figure S2. The HRTEM image itself is shown in figure 3b of the main text and was chosen for this comparison due to the low convergence angles possible with this technique. Consequently, the transmitted wave vectors appear as sharp spots in the power spectrum allowing for the observance of weaker, intermediate Bragg reflections that may be diluted in CBED data.

Figure S2a shows $R_{\mathrm{Magnetite}}(u,v)$ $[\bar{1}\ \bar{1}\ 1]$. Figure S2d shows $R_{\mathrm{Fe}}(u,v) \otimes R_{\mathrm{Magnetite}}(u,v)$ in blue overlaid on the Fourier-transformed HRTEM image. The match between theory and experiment is excellent. The Fe_3O_4 (0 4 4) plane matches well with Fe (0 0 2) while the Fe_3O_4 (4 2 2) planes are offset by the correct amount to generate the observed $\mathbf{\Delta g}$ vector. Additional reflections such as Fe_3O_4 (2 2 4) and (2 0 2) fully explain the presence of the three distinct "rows" of reflections between (0 0 0) and Fe (0 0 2), and the expected intensities even match very closely with experiment, with any discrepancies being easily accounted for by the uncertainty in the thickness of the individual layers and the choice of scattering model in JEMS.

Figure S2b shows $R_{\mathrm{Maghemite}}(u,v)$ $[\bar{1}\ \bar{1}\ 1]$. It is immediately clear that, despite the similar crystal structure, Maghemite exhibits a significantly different transmittance function compared to Magnetite in this orientation, with twice as many rows observed along the [0 4 4] direction (denoted with the white arrows in figure S2e). Consequently, $R_{\mathrm{Fe}}(u,v) \otimes R_{\mathrm{Maghemite}}(u,v)$

does not fit well with the experimental observations, as seen in figure S2e. The appearance of these extra rows can be understood by considering the charge balance of the oxide structure. In Maghemite, all of the iron present is trivalent, resulting in a charge inbalance.[5] This inbalance is compensated for by the addition of vacancies on the cation sites resulting in a reduction of the symmetry. Consequently, reflections in the diffraction pattern that are kinematically forbidden in Magnetite appear in Maghemite. For the argument in favor of Maghemite to be convincing, one would expect seven distinct "rows" of reflections between (0 0 0) and Fe (0 0 2) to appear in the Fourier-transformed HRTEM image, but this was never observed. If the oxide is described as the disordered structure $Fe_{3-\delta}O_4$, the strength of these additional reflections will increase with increasing δ. Their absence in all of the FFT or CBED patterns indicates that δ must be close or equal to 0.

Figure S2c shows $R_{\text{Wüstite}}(u,v)$ $[\bar{1}\ \bar{1}\ 1]$. Also here, despite the similar structure, it is clear that this orientation reveals significant structural differences with Magnetite, with half as many rows observed along the [0 4 4] direction. When the convolution between $R_{\text{Wüstite}}(u,v)$ and $R_{\text{Fe}}(u,v)$ is overlaid with the Fourier transformed HRTEM data in figure S2f, it is quite clear that this oxide lacks the necessary structure to adequately explain all of the observed spatial frequencies. We note here that this analysis considers only stoichiometric FeO. Modulated FeO structures are known to arise when iron is deficient, resulting in a structural formula $Fe_{1-x}O$. However, none of the published structures of this phase that we investigated fit well with the experimental data. For example, Yamamoto publish $Fe_{1-x}O$ where $x = 0.098$.[10] In this case, five additional rows of reflections between (0 0 0) and Fe (0 0 2) are observed. However, this symmetry does not match with the observed data, which would require three additional rows. Thus neither stoichiometric nor modulated Wüstite can adequately explain the experimentally observed reflections.

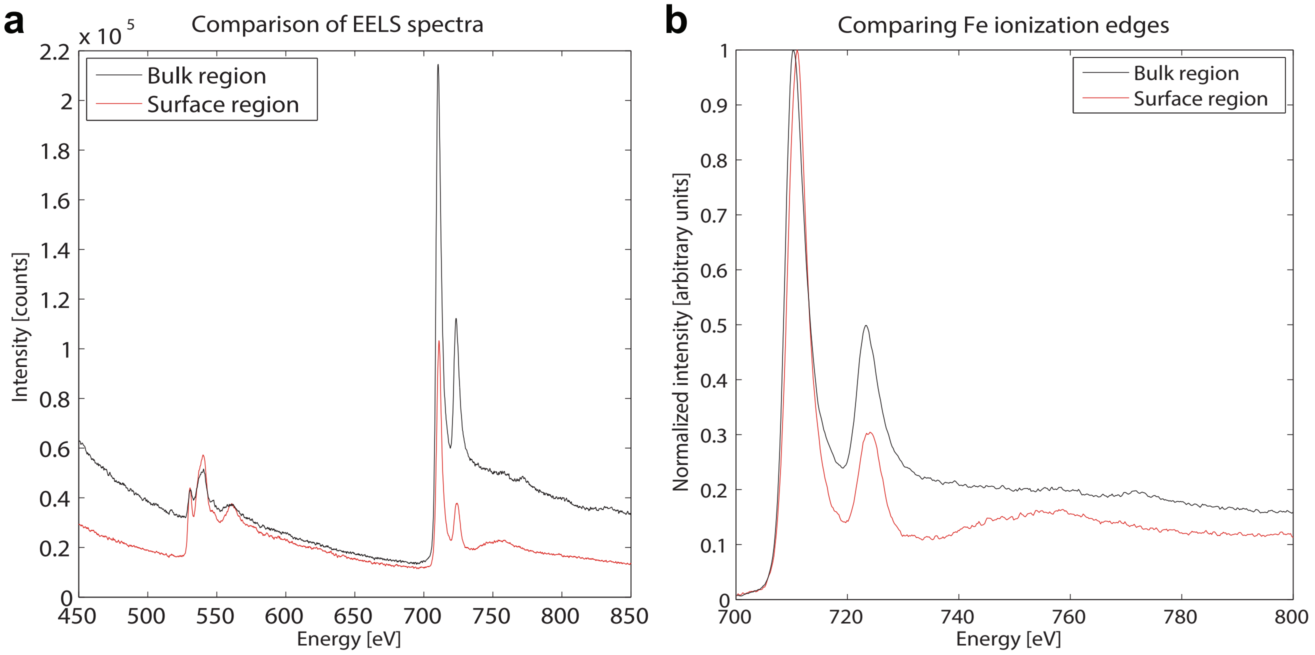

Figure 3. (a) Summation over the raw core-loss EELS spectra from the bulk (black) and surface (red) regions. (b) Same data as (a) but with the pre-edge background removed and scaled to the energy range 700 - 800 eV. The intensities are also normalized to the max value of each spectra to facilitate comparison of the white line ratio.

To conclude this section, we discuss the quantitative core-loss EELS data shown in figure 1 of the main text. As discussed, this experimental design allows for spatial segregation of regions containing different metallic iron and iron oxide compositions. A comparison of the on-axis (non-magnetic) EELS data is presented in figure S3. Each of these spectra represents a summation over 45 individual spectra located within the film (black line) as well as near the film surface (red line). Figure S3a shows the untreated spectra from these regions while figure S3b compares the iron ionization edges after removal of the pre-edge background and normalization to the max value in each spectra. The spectra from these different spatial regions reveal distinct shapes with a significantly different white line ratio and even a chemical shift of 1.5 eV is observed. From this, we can conclude that the "surface" region consists primarily – if not entirely – of iron oxide.

The ratio Fe:O in this surface region is also shown in figure 1 of the main text and averages to 0.424 ± 0.038, close to what would be expected if only Fe_3O_4 was present. If FeO or $Fe_{1-x}O$ were present, we would expect this ratio to be significantly reduced. This value is also more consistent with Fe_3O_4 than γ–Fe_2O_3; however, as discussed in the manuscript, the magnitude

of the systematic error bar is too uncertain to make this claim with high confidence. This distinction is best made using the structural data presented above.

Taking all of these observations into consideration, we feel that the oxide cladding layers are most accurately described as $Fe_{3-\delta}O_4$ with δ close to 0, which is consistent with the description of an Fe (1 1 0) surface under comparable preparation conditions.[3,4]

Derivation of oxide thickness

The thickness of the oxide layer was derived in the following manner. An electron probe of radius r illuminates the lamella with a total thickness t_{tot}. The thickness of the oxide is t_{ox}. The oxide is assumed to be Fe_3O_4, and the justification for this assumption is provided in the manuscript text as well as the section above. In this case, the density of atoms in Fe_3O_4, ρ_{ox}, is 103.5 atoms/nm^3. The density of iron atoms in metallic iron, ρ_{Fe}, is 84.95 atoms/nm^3. Neglecting beam spread, the total volume of Fe_3O_4, V_{ox}, and metallic iron, V_{Fe}, is thus

$$V_{ox} = \pi r^2 t_{ox} \ , \tag{3}$$

$$V_{Fe} = \pi r^2 (t_{tot} - t_{ox}) \ . \tag{4}$$

The total number of atoms in the oxide layer is given by N_{ox}, where $N_{ox} = \rho_{ox} V_{ox}$. The numbers of iron and oxygen atoms in the oxide layer are given by $N_{Fe,ox}$ and N_O, respectively. As a consequence of the assumption of Fe_3O_4 for the oxide,

$$N_{Fe,ox} = \frac{3}{7} N_{ox} \ \text{and} \ \mathrm{N_O} = \frac{4}{7} \mathrm{N_{ox}} \ . \tag{5}$$

It is assumed that all of the oxygen detected in the EELS measurements belongs to N_O.

The ratio of the number of iron atoms to oxygen atoms is given as

$$R = \frac{N_{Fe,tot}}{N_O} \ . \tag{6}$$

R is the experimental input and its value for both iron and oxygen is presented in figure 1 of the text. As R is extracted from the Digital Micrograph software package using the quantitative EELS module, the influence of experimental parameters such as collection angle and acceleration voltage on the detected scattering cross section of these two species is considered. It can thus be assumed that R is a reasonably accurate representation of the atomic ratio. Hence

$$R = \frac{N_{Fe,ox} + N_{Fe,metal}}{N_O} = \frac{\frac{3}{7}\rho_{ox} V_{ox} + \rho_{Fe} V_{Fe}}{\frac{4}{7}\rho_{ox} V_{ox}} \ . \tag{7}$$

Substituting V_{ox} and V_{Fe} and solving for t_{ox}, one finds

$$t_{ox} = \frac{t_{tot}\rho_{Fe}}{\frac{4R}{7}\rho_{ox} - \frac{3}{7}\rho_{ox} + \rho_{Fe}} \ . \tag{8}$$

Error propagation in the application of sum rules

The calculation of m_L/m_S is well known to be highly sensitive to the experimental conditions, inducing non-negligible uncertainties in the end result. Here, we present our method for estimating the error of the calculations. This will be treated in two parts. First, the statistical error due to noise as well as its propagation through the application of the sum rules is discussed. Second, the systematic error introduced by the choice of background normalization window is described.

Statistical error in the calculations arises from a number of sources, and we have attempted to account for as many of them as possible in addition to propagating them through the entire analysis.[11] First, the errors arising from counting statistics were quantified assuming the counts follow Poisson's distribution. Second, the errors due to the pre-edge background fitting routine were quantified by calculating the one sigma prediction interval of the background fit over the energy range used for quantification. Both of these error vectors for both Chiral Plus and Chiral Minus spectra were added in quadrature when the background subtraction was performed. The errors on the Chiral Minus spectra were added to the uncertainty in calculating the normalization factor. Subsequently, the error vectors for both spectra were again added in quadrature when the difference was

computed to extract the EMCD signal. This error vector was used to compute the cumulative error for the integrals over which the sum rules were computed. Finally, this error was propagated through the sum rules formula.

We observe that, for the data presented in this work, the absolute statistical m_L/m_S error typically returned is around 0.01. This is largely independent of the absolute value of m_L/m_S, but we note that this dramatically increases when fewer spectra are used for the summation, causing the noise level to go up. The source of this increase appears to be primarily due to the uncertainties in the background extrapolation, which dominates the other error sources by approximately three orders of magnitude.

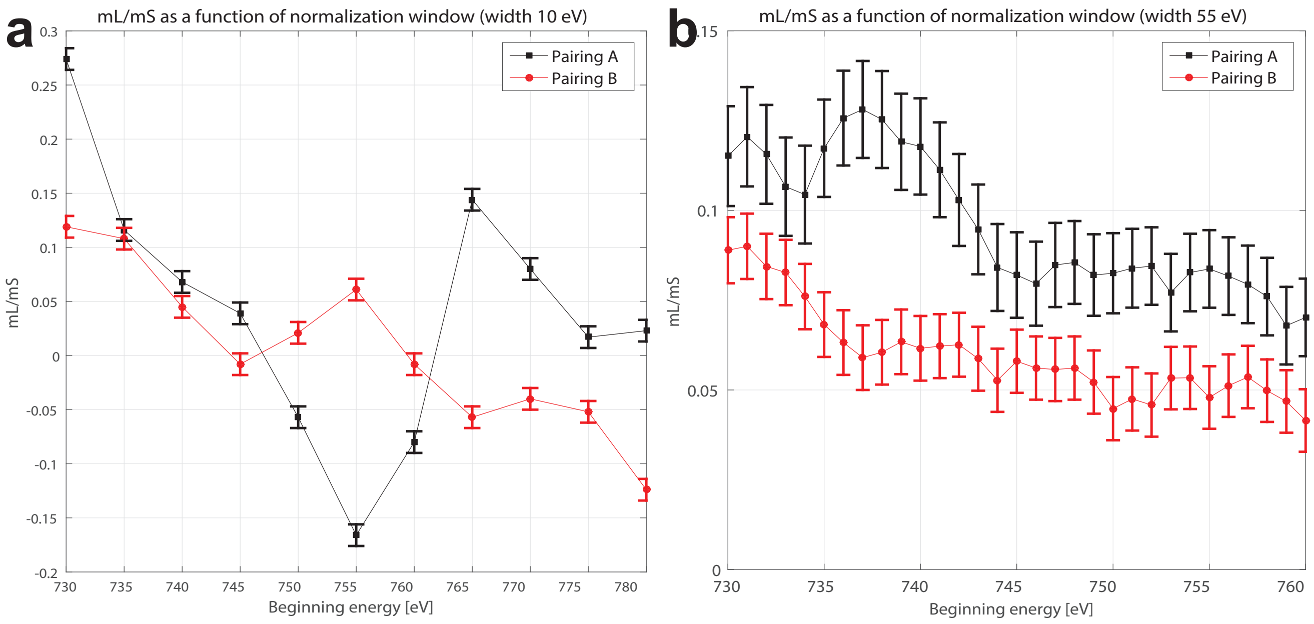

Figure 4. Extracted m_L/m_S values using a normalization window of (a) 10 eV and (b) 55 eV. The statistical error calculated for each value is given as an error bar. Note the different y-axis scales.

An additional error source not accounted for by the statistical treatment above stems from the choice of the position and width of the post-edge normalization window. The intensity variations in the EMCD signal integral in the post-edge region are partially correlated, likely due to the failure to remove all of the correlated noise in the acquisition. Accordingly, significant variations can take place over a range of some 5 eV, meaning that smaller window widths will return highly variable results. This can be clearly seen in figure S 4a, where a 10 eV window was used for the normalization at a variety of energies. Using this normalization window width results in wild fluctuations of m_L/m_S, with a number of results returning negative values. For each individual calculation, the statistical error was calculated using the error propagation method described above and is provided in the form of an error bar. Critically, this error appears to be significantly smaller than the variation of m_L/m_S over the post-edge normalization range, which could easily lead to false sense of security when reporting quantitative results.

To mitigate these systematic errors, we have chosen to use a large normalization window of 55 eV. This width is larger than the fluctuations from correlated noise in the post-edge region, thereby reducing their influence. To assess this effect, we have varied the position of the window in increments of 1 eV and recorded the value of m_L/m_S each time along with the statistical error. The results of this analysis are shown in figure S 4b. A dramatic decrease in the fluctuation of the values is observed, suggesting that this method leads to more consistent measurements. An additional observation is that there appears to be a slight downward trend of m_L/m_S when the normalization window begins at lower energies. This trend appears to stabilize for starting energies of around 745 eV. If we consider all of the data points in figure S 4b above, $m_L/m_S = 0.096 \pm 0.018$ and 0.060 ± 0.013 for aperture pairings A and B, respectively. If, however, we only consider the values after 745 eV, then m_L/m_S becomes 0.085 ± 0.012 and 0.053 ± 0.006 for aperture pairings A and B, respectively. One justification for choosing an energy range further away from the iron edges comes from a comment claiming that magnetic EXELFS may influence the magnetically dichroic signal to some extent in the post-edge region.[12] Accordingly, as our estimate of uncertainty in the manuscript, we have considered both the statistical and systematic errors, adding them in quadrature with equal weights.

Overview of the TEM lamella

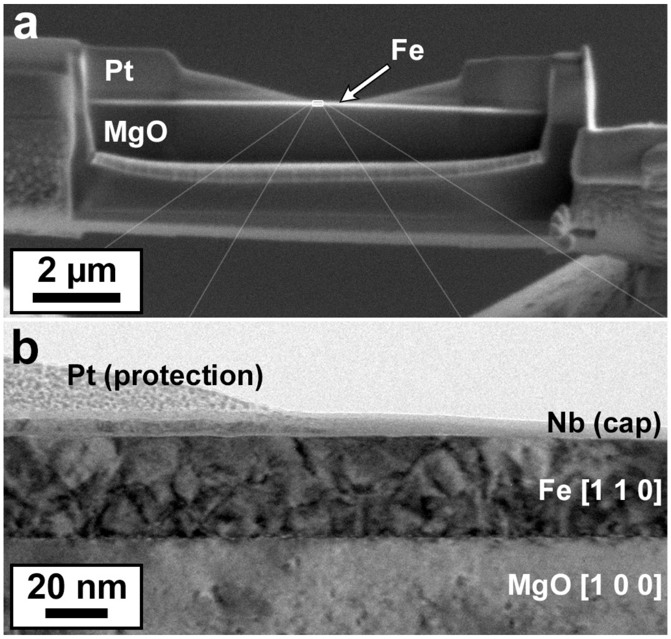

Figure 5. a) Overview micrograph of the prepared iron thin film cross-section generated using secondary electrons in the FIB instrument. The region of interest for the EMCD measurements is shown in a small white box and enlarged below. b) Bright-field TEM micrograph acquired with the sample tilted so that the beam is nearly parallel to the MgO [1 0 0] direction.

In figure S5a, the as-polished lamella is illuminated with 3 kV electrons and contrast is generated using a secondary electron detector mounted within the FIB. The iron thin film in this image appears as a bright contrast. In the middle of the lamella - denoted by the arrow - some regions were polished to a thickness of less than 22 nm, as determined by EELS $t \cdot \lambda^{-1}$ measurements (see figure 1 of the main text). A bright field TEM image of part of this thin region is shown in figure 5b. This image was generated by tilting the lamella to near MgO [1 0 0] and inserting the objective aperture under nearly parallel illumination conditions. The iron film contains numerous regions where the contrast is darkened corresponding to a certain degree of crystallographic disturbance, likely caused the presence of a surface oxide.

References

1. Leygraf, C. and Ekelund, S. A LEED-AES study of the oxidation of Fe (110) and Fe (100). *Surf Sci.* **40**, 609–635 (1973).
2. Davies, R. *et al.* The oxidation of Fe(111). *Surf Sci.* **605**, 1754 – 1762 (2011).
3. Kim, H.-J., Park, J.-H., and Vescovo, E. Fe_3O_4(111)/Fe(110) magnetic bilayer: Electronic and magnetic properties at the surface and interface. *Phys. Rev. B* **61**, 15288–15293 (2000).
4. Kim, H.-J., Park, J.-H., and Vescovo, E. Oxidation of the Fe (110) surface: An Fe_3O_4 (111) / Fe (110) bilayer. *Phys. Rev. B* **61**, 15284–15287 (2000).
5. Cornell, R. M. *The iron oxides: structure, properties, reactions, occurrences, and uses*. (Wiley-VCH, Weinheim, 2003).
6. Pellegrin, E. *et al.* Characterization of Nanocrystalline γ-Fe_2O_3 with Synchrotron Radiation Techniques. *Phys. Status Solidi B* **215**, 797–801 (1999).
7. Amidror, I. *The theory of the Moiré phenomenon*. Number 38 in Computational imaging and vision. (Kluwer Academic, Dordrecht, 2009).
8. Stadelmann, P. A. EMS - a software package for electron diffraction analysis and HREM image simulation in materials science. *Ultramicroscopy* **21**, 131–145 (1987).
9. Peng, L. M. Electron atomic scattering factors and scattering potentials of crystals. *Micron* **30**, 625–648 (1999).
10. Yamamoto, A. Modulated structure of wustite ($Fe_{1-x}O$) (three-dimensional modulation). *Acta Cryst.* **38**, 1451–1456 (1982).

11. Bevington, Philip R. *Data reduction and error analysis for the physical sciences*. (McGraw-Hill, 2003).

12. Goering, E., Lafkioti, M., and Gold, S. Comment on “Spin and Orbital Magnetic Moments of Fe_3O_4”. *Phys. Rev. Lett.* **96** (2006).
